# Oxidative Stress and Inflammation in Diabetic Complications

**DOI:** 10.1155/2014/679754

**Published:** 2014-05-04

**Authors:** Ajit Vikram, Durga Nand Tripathi, Ashutosh Kumar, Sandeep Singh

**Affiliations:** ^1^Department of Internal Medicine, Carver College of Medicine, University of Iowa, IA 52246, USA; ^2^Centre for Translational Cancer Research, IBT, Texas A&M HSC, Houston, TX 77030, USA; ^3^Department of Pharmacology and Toxicology, National Institute of Pharmaceutical Education and Research, Hyderabad, Andhra Pradesh 500037, India; ^4^Centre for Genetic Diseases and Molecular Medicine, Central University of Punjab, Mansa Road, Bathinda, Punjab 151001, India

The guest editors of this issue are pleased to present this compendium of research and review articles focusing on the role of oxidative stress and inflammation in diabetes and diabetes-associated complications. Increased prevalence of insulin-resistance, a prediabetic condition, and type 2 diabetes is a major health concern all over the world. As per WHO estimates, it is expected that by 2030 the number of patients with diabetes will be more than double. Progressive deterioration in metabolic control with existing therapeutic modalities necessitates better understanding and newer therapeutic interventions for the effective management of diabetes.

Oxi-flammation (oxidative stress and inflammation) affects a multitude of cellular responses in various organ systems, and progression of insulin-resistance is known to be associated with chronic systemic inflammation and increased oxidative stress. The positive feedback cycle involving chronic systemic inflammation, oxidative stress, and progression of insulin-resistance contributes to several diabetes-associated complications, including cardiovascular diseases, nephropathy, neuropathy, retinopathy, urological diseases, and cancer [1–3]. Impairment in insulin synthesis, release, and/or action (insulin-resistance), a hallmark of diabetic condition, results in several secondary conditions. Decreased insulin-sensitivity is often accompanied by compensatory rise in the insulin level, posing an extra burden on pancreatic *β*-cells. In addition to maintaining plasma glucose level, insulin has a growth-stimulating effect. The IRS/PI3-Kinase dependent downstream signaling of insulin is primarily concerned with glucose uptake and metabolic effects, whereas MEK/ERK dependent signaling is responsible for its mitogenic action. Resistance does not develop equally to metabolic and mitogenic signaling of insulin [4], and therefore hyperinsulinemia results in overactivation of mitogenic signaling and has adverse effects on different tissue systems. Increased inflammation, oxidative stress, dyslipidemia, and glucotoxicity with an extra workload on *β*-cells eventually meet a point where *β*-cells are no longer able to meet the ever increasing demand of insulin, resulting in the development of frank diabetes (Figure 1). In this special issue, K. Batumalaie et al. report that gelam honey regulates the expression and/or activation of insulin-receptor signaling mediators and improves cellular insulin content in HIT-T15 cells (pancreatic *β*-cells) exposed to hyperglycemic condition.

Insulin-resistance promotes endothelial dysfunction and it is an independent risk factor for the development of coronary artery diseases. The review article embodied in this issue by S.I.Q.S. Ikmal et al. describes potential biomarkers of insulin-resistance and atherosclerosis in type 2 diabetic patients with coronary artery disease. J. Fuentes-Antrás et al. believe that hyperglycemia, dyslipidemia, and insulin-resistance are among the most important factors which contribute to low-grade inflammation in the diabetic heart. In this issue, their review article “*Activation of toll-like receptors and inflammasome complexes in the diabetic cardiomyopathy-associated inflammation*” suggests that toll-like receptors and inflammasome-complexes may be key inducers for inflammation probably through nuclear factor-kappa B (NF-*κ*B) activation and oxidative stress. However, the peroxisome proliferator-activated receptors and mammalian life-span regulator sirtuins may be the potential therapeutic targets in mitigating both toll-like receptors and inflammasome signaling. The review article by R. Sandireddy et al. outlined futuristic strategies targeting oxidative stress and neuroinflammation in diabetic neuropathy. The authors are of opinion that a combinatorial approach targeting multiple signaling pathways might be of practical use in combating diabetic neuropathy.

Heatstroke is a medical emergency condition and can have profound deleterious effect on brain and other tissue systems in body. As diabetes is characterized by deregulation of metabolic control, the patients are more likely to fall short in handling stress conditions, including heat stress. Although, the association between diabetes and heatstroke is not very clear and further studies are required, animal studies by C.-C. Hsu et al. suggest that diabetic condition augments the deleterious effects of heat stress on body temperature regulation and cerebral blood flow and is associated with neuronal death in hypothalamus. The authors have further reported that these conditions are at least partially ameliorated with the intervention of Chinese herb Honokiol.

M. Roehrs et al. reported improved glucose and lipid profile in response to Bixin, a annotto carotenoid, in streptozotocin (STZ) induced diabetic model. Moreover, Bixin treatment restored antioxidant enzyme super oxide dismutase activity and reduced oxidative stress as evident from decreased protein oxidation and nitric oxide production. On the other hand, the Norbixin, a relatively less lipophilic analogue of Bixin, was less or not effective in inducing these effects.

An increase in body fat is generally associated with increased risk of metabolic diseases such as type 2 diabetes mellitus. Body mass index (BMI) criteria are currently the primary focus in metabolic disorder treatment recommendations. S. Kaštelan et al. reported a parallel association between BMI and diabetic retinopathy.

Taurine, one of the most abundant amino acids in the mammalian organs, is known to have beneficial effects on experimental diabetic nephropathy. Here, authors have shown that taurine-mediated improvement in diabetic nephropathy could be attributed to its antioxidant property and amelioration of diabetes-induced increase or decrease in VEGF or Nephrin expression. Taurine-treated group also showed reduced reactive oxygen species levels indicating that it possibly inhibits the progression of diabetic nephropathy by strengthening antioxidant defense.

In conclusion, the natural products hold high promise to provide lead for the development of therapeutically relevant molecules. Perhaps, in contrast to the usual analytical approach, a synthetic approach where a complex mixture of molecules, which is often the case with natural products, may help us to answer complex disease conditions such as diabetes and diabetes-associated complications. Understanding of early and effective biomarkers and use of suitable protective/preventive strategies might open new therapeutic avenues for patients suffering from diabetes and its associated complication. We believe that the present issue with reviews and research articles has well summarized current development in the field and will be of interest to the readers.

*Ajit Vikram* *Ajit Vikram* *Durga Nand Tripathi* *Durga Nand Tripathi* *Ashutosh Kumar* *Ashutosh Kumar* *Sandeep Singh* *Sandeep Singh*

## Figures and Tables

**Figure 1:**
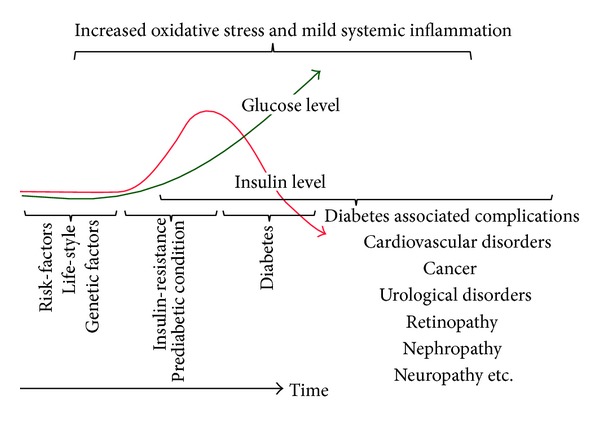
Life-style and genetic factors contribute to the development of oxidative stress and mild systemic inflammation resulting in decreased insulin-sensitivity which is accompanied by compensatory hyperinsulinemia and mild hyperglycemia, a prediabetic state. As *β*-cells fail to meet increased demand for insulin, frank diabetes develops with subnormal level of insulin and even higher glucose level. Hyperinsulinaemic condition in prediabetic state and profound dysregulation of glucose and lipid metabolism in diabetic condition further aggravate oxidative stress and inflammation. All these factors contribute to the development of diabetes associated complications.
